# Colchicine ameliorates the chronic inflammatory state in patients with chronic rheumatic valvular heart disease: a pilot study

**DOI:** 10.1186/s43044-020-00080-2

**Published:** 2020-07-16

**Authors:** Osama Rifaie, Mahmoud Badr, Ahmed Abdel Salam, Haitham Galal

**Affiliations:** Department of Cardiology, Faculty of Medicine, Ain Shams University, Cairo, Egypt

**Keywords:** Rheumatic fever, Rheumatic heart disease, Colchicine, Inflammatory markers, C-reactive protein, Interleukin-6

## Abstract

**Background:**

Inflammation is an important contributor to the pathogenesis of rheumatic heart disease (RHD). High serum levels of C-reactive protein (CRP) and interleukin-6 (IL-6) are commonly seen in patients with chronic (RHD) and indicate the presence of a chronic inflammatory state. The aim of this study was to assess the effect of colchicine as anti-inflammatory drug on the serum levels of the inflammatory markers (CRP) and (IL-6) in patients with chronic (RHD).

**Results:**

This is a prospective controlled study that enrolled thirty-five patients with chronic (RHD) visiting Ain Shams University Hospital’s outpatient clinic for receiving regular long acting penicillin as rheumatic fever prophylaxis. Ten matched healthy individuals were taken as control group. Blood samples for serum levels of CRP and IL-6 were collected before and 1 month after receiving colchicine 0.5 mg BID. Mean (CRP) level was 6.09 ± 4.39 IU/ml versus 0 IU/ml in the control group respectively (*P* = 0.0001). Mean (IL-6) level was 113.57 ± 37.41 ng/l versus 10.50 ± 5.99 ng/l, in the control group (p = 0.0001). Mean (CRP) was 6.09 ± 4.39 IU/ml before and became 3.34 ± 3.07I U/ml 1 month after colchicine therapy. Mean (IL-6) level was 113.57 ± 37.4 ng/l before and became 45.57 ± 20.39 ng/l 1 month after colchicine therapy (*P* = 0.001).

**Conclusion:**

In this pilot study**,** using colchicine as anti-inflammatory drug in patients with chronic (RHD) significantly reduced the serum inflammatory markers (CRP) and (IL-6), thus helping in ameliorating their chronic inflammatory state.

## Background

Rheumatic heart disease (RHD) is a systemic autoimmune disorder related to prior streptococcal infection. It is the leading cause of acquired heart disease in those under the age of 40 years living in developing nations [1]. The incidence of rheumatic fever (RF) and prevalence of rheumatic heart disease vary substantially among countries. RHD is associated with a progressive inflammatory reaction that leads to variable degrees of cardiac valves lesions and may progress to severe degrees leading to surgical interventions and sometimes ultimately deadly outcomes [1].

Despite the pathophysiology of RHD is well known to be due to an immune-mediated mechanism initiated by the molecular mimicry, the underlying mechanisms involved in the disease progression are not completely understood [2]. The rate of progression of valvular lesions may be influenced by multiple factors, especially genetic predisposition and host’s immune response. The cytokines involved in the activation of the immune response may play a role in determining RHD severity [2, 3]. In fact, it has been shown that there is an ongoing inflammatory state in patients with RHD evidenced by high serum levels of some inflammatory markers, especially IL6 and CRP [4–6]. This study was done to assess the effect of colchicine as anti-inflammatory drug on serum levels of inflammatory markers CRP and IL-6 in patient with RHD, thereby possibly ameliorating the chronic inflammatory state in chronic RHD.

## Methods

This was a prospective controlled study involving a total number of forty-five participants. The treatment group comprised 35 patients with chronic RHD visiting Ain Shams University Hospital’s outpatient clinic for receiving long acting benzathine penicillin as RF prophylaxis. The control group was formed of matched ten healthy individuals not known to have rheumatic heart diseases or suffering from any other disease that might cause raising of inflammatory markers.

The following patients were excluded from the study: Rheumatic heart patients with prosthetic valves, any patient with other inflammatory process rather than RHD, patients with atrial fibrillation, and patients who have medical disorders listed as contraindication for colchicine treatment as severe renal and hepatic disorders.

*All the patients were subjected to the following:*

1. Medical history was taken in details from all patients with focus on the following:

- Medical history of past and current inflammatory conditions that can lead to increase in levels of inflammatory mediators.
- Risk factors for rheumatic fever including age, socioeconomic class, family history of rheumatic fever, recurrent streptococcal infection, and previous attacks of rheumatic fever.
- Patient compliance to long acting penicillin and type of regimen.

2- General and local cardiac examination was done for all patients.
3- Transthoracic echocardiography done for all patients within 6 months to document and estimate the type and severity of valvular affection.
4- Detailed informed consent was taken from each patient.
5- Venous samples were drawn from all cases under complete aseptic conditions. Blood was centrifuged, and 3 ml serum were collected then stored at – 20 °C for further analysis. Serum levels of CRP and IL-6 were measured before receiving colchicine treatment at Ain Shams University Hospital immunology laboratories. Serum samples were analyzed for IL-6 and CRP using stat fast 1100 Elisa technique (Human IL-6 KITS). CRP was measured by the semi quantitative latex technique.

The 35 RHD patients received 0.5 mg of colchicine BID daily for 1 month, after which measurements of serum IL-6 and CRP were repeated.

The study protocol was approved by the research committee of our institution after obtaining informed consents from all of the patients and the control group. The study complies with the declaration of Helsinki.

### Statistical methods

Data were coded and entered using the statistical package SPSS version 25, Chicago. Illinois. Data were summarized using mean, standard deviation, and minimum and maximum for quantitative variables. Frequencies (number of cases) and relative frequencies (percentages) were used for categorical variables. Comparisons between groups were done using unpaired *t* test*.* For comparing categorical data, chi-square (*χ*^2^) test was performed. One-way ANOVA test and post hoc analysis (LSD method) were used to compare the serum levels of IL6 and CRP in the three regimens of long acting penicillin. Fischer’s exact test was used instead when the expected frequency is less than 5*. P* values less than 0.05 were considered as statistically significant.

## Results

### Basic demographic and clinical data

No significant difference was found regarding age and gender between patients and the control group. Patients’ mean age was 33.77 ± 4.83 years (ranging from 28 to 54 years), while the mean age of the control group was 31.70 ± 2.98 years (ranging from 29 to 38), *P* = 0.2. In the patient group, 24 (68.6%) were females and 11 (31.4%) were males. In the control group, 5 (50%) were females and 5 (50%) were males (*P* = 0.279). Serum CRP was zero (0) IU/ml in the control group versus 6.09 ± 4.39 IU/ml in the patient group (*P* < 0.0001). Serum IL-6 was 10.50 ± 5.99 ng/l in the control group versus 113.57 ± 37.41 ng/l in the patient group (*P* < 0.0001) (Table 1).

**Table 1:** Baseline data of patient and control groups before receiving colchicine

		Control group	Patients group	Test value	P-value	Sig.
		No. = 10	No. = 35			
Age (years)	Mean ± SD	31.70 ± 2.98	33.77 ± 4.83	− 1.283	0.206	NS
	Range	29 – 38	28 – 54			
Gender	Female	5 (50.0%)	24 (68.6%)	1.171	0.279	NS
	Male	5 (50.0%)	11 (31.4%)			
CRP level (IU/ml)	Mean ± SD	0 ± 0	6.09 ± 4.39	− 4.343	0.0001	HS
	Range	0–0	0–12			
IL-6 level (ng/L)	Mean ± SD	10.50 ± 5.99	113.57 ± 37.41	− 8.612	0.0001	HS
	Range	5–20	30–160			

*SD* standard deviation, *CRP* C-reactive protein, *IL-6* interleukin-6, *NS* non-significant, *HS* highly significant

Table 2 shows the echocardiographic data of RHD patients. Seventeen patients (48.5%) had mild mitral regurgitation (MR), 4 (11.4%) had moderate (MR), 5 (14.2%) had mild aortic stenosis, 4 (11.4%) had mild tricuspid regurgitation, 1 (2.8%) had moderate tricuspid regurgitation, 4 (11.4%) had mild mitral stenosis, and 1 patient (2.8%) had severe mitral stenosis.

**Table 2:** Echocardiographic data of valvular lesions in the patient group

		Total no. = 35
Mitral regurgitation	Negative	14 (31.1%)
	Mild	17 (48.5%)
	Moderate	4 (11.4%)
	Severe	0 (0.0%)
Aortic stenosis	Negative	30 (85.7)%)
	Mild	5 (14.2%)
	Moderate	0 (0.0%)
	Severe	0 (0.0%)
Tricuspid regurgitation	Negative	30 (85.7%)
	Mild	4 (11.4%)
	Moderate	1 (2.8%)
	Severe	0 (0.0%)
Mitral stenosis	Negative	30 (85.7%)
	Mild	4 (11.4%)
	Moderate	0 (0.0%)
	Severe	1 (2.8%)

All of the patients were on penicillin as prophylaxis, with a mean duration of 4.77 ± 4.7 years (ranging from 1 to 20 years). Fifteen patients (42.8%) received penicillin prophylaxis every 15 days. Fourteen patients (42%) received it every 21 days. Only 6 patients received it every 30 days. The mean timing was every 19.97 ± 5.39 days (ranging from 15 to 30 days).

**Fig. 1:**
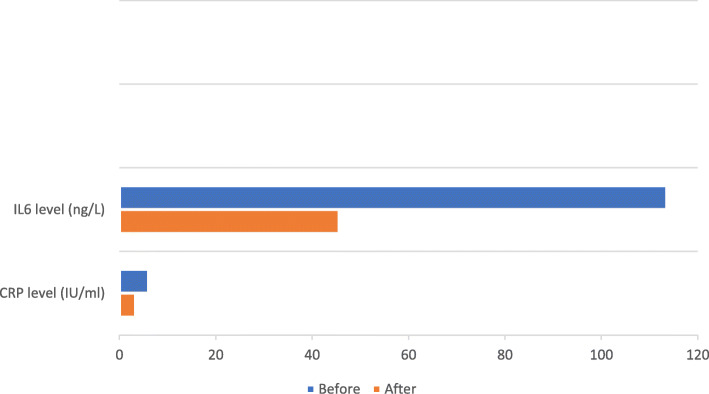
Serum IL-6 and CRP before and after receiving colchicine treatment in the patient group

After 1 month of receiving colchicine 0.5 mg twice daily as an anti-inflammatory treatment, patients showed highly significance reduction in the serum level of serum inflammatory markers IL-6 and CRP as shown in Table 3 and Fig. 1.

**Table 3:** Serum IL-6 and CRP before and after receiving colchicine treatment in the patient group

		Before	After	Test value	***P*** value
CRP level (IU/ml)	Mean ± SD	6.09 ± 4.39	3.34 ± 3.07	6.099	0.0001
	Range	0–12	0–12		
IL-6 level (ng/L)	Mean ± SD	113.57 ± 37.41	45.57 ± 20.39	12.501	0.0001
	Range	30–160	25–100		

*CRP* C-reactive protein, *IL-6* interleukin-6, *SD* standard deviation

Table 4 shows the impact of the different regimens of long acting penicillin prophylaxis on serum inflammatory markers before and after receiving colchicine 0.5 mg twice daily for 1 month. Using one-way ANOVA test, the three different penicillin regimens showed significant reduction in IL-6 and CRP serum levels after receiving colchicine treatment. However, on using post hoc analysis, we found that serum levels of IL-6 and CRP were significantly higher before and after colchicine therapy in the 30-day than in 15-day regimen. However, CRP but not IL-6 levels were significantly higher in the 30-day versus 21-day regimens. Moreover, there was no difference between the 21- and the 15-day regimens as regards the level of IL-6 and CRP whether before or after colchicine (Table 5).

**Table 4:** Effect of colchicine on the serum IL-6 and CRP in relation to different penicillin regimens

		Penicillin timing (days)			Test value	P value*	Sig.
		15	21	30			
CRP level before starting colchicine (IU/ml)	Mean ± SD	4.80 ± 4.51	5.36 ± 3.56	11.00 ± 2.45	5.909	0.007	HS
	Range	0–12	0–12	6–12			
CRP level after 1 month of regular colchicine intake (IU/ml)	Mean ± SD	2.20 ± 2.65	3.00 ± 2.63	7.00 ± 2.45	7.435	0.002	HS
	Range	0–6	0–6	6–12			
IL-6 level before starting colchicine (ng/L)	Mean ± SD	98.67 ± 32.15	117.50 ± 42.73	141.67 ± 14.72	3.373	0.047	S
	Range	30–145	40–160	120–160			
IL-6 level after 1 month of regular colchicine intake (ng/L)	Mean ± SD	36.67 ± 11.75	49.29 ± 22.26	59.17 ± 25.58	3.423	0.045	S
	Range	25–60	25–100	35–100			

*CRP* C-reactive protein, *IL-6* interleukin-6, *SD* standard deviation

*P value between the 15-, 21-, and 30-day regimens of long acting penicillin. HS = *P* value < 0.0001. S = *P* value < 0.001

**Table 5:** *P* value using post hoc analysis by LSD method for comparing IL6 and CRP levels in the 3 penicillin regimens

	15 Vs 21	15 Vs 30	21 Vs 30
CRP level before starting colchicine (IU/ml)	0.701 (NS)	0.002	0.005
CRP level after 1 month of regular colchicine intake (IU/ml)	0.416 (NS)	0.001	0.004
IL6 level before starting colchicine (ng/L)	0.158 (NS)	0.016	0.167 (NS)*
IL6 level after 1 month of regular colchicine intake (ng/L)	0.085 (NS)	0.020	0.296 (NS)*

*CRP* C-reactive protein, *IL-6* interleukin-6

*(NS) = *P* value > 0.05. One-way ANOVA test followed by post hoc analysis by LSD test

In the patient group, only one patient experienced mild abdominal pain which subsequently disappeared while still on colchicine; no other side effects were noted.

## Discussion

The presence of ongoing chronic inflammation in chronic RHD is well known. Some studies have shown that it is related to the development of valvular lesions [4]. In fact, some authors [4, 5] found that the serum levels of inflammatory markers were correlated to the severity of the valvular lesions. Davutoglu et al. showed that chronic RHD was associated with high serum inflammatory mediators which correlated strongly with the severity of valve lesion, valve scarring, subsequent valve calcification, and decreased functional status. They recommended that further research was needed to indicate whether anti-inflammatory drugs might reduce progression of the valvular lesions as well as morbidity and mortality in patients with chronic RHD [4].

In our study, we investigated the effect of colchicine as anti-inflammatory drug on the serum levels of inflammatory markers CRP and IL-6 In chronic RHD. To our knowledge, this is the first study to address the effect of colchicine as anti-inflammatory drug on the serum levels of inflammatory markers CRP and IL-6 in chronic RHD. The baseline of the serum inflammatory markers CRP and IL-6 before starting the colchicine treatment showed significant difference between the patients and the control group. This confirms the presence of the ongoing inflammation in chronic RHD patients, in accordance with other studies [4–6].

Soares and his colleagues investigated the cytokine plasma levels in patients with chronic RHD as possible markers for the severity of the disease; the study concluded that high levels of inflammatory cytokines were correlated with the severity of RHD. Moreover, the co-regulated expression of IL-6 and TNF-α was associated with a worse clinical presentation [7]. On looking at our results, patients showed highly significant reduction in the serum inflammatory markers after 1 month of receiving colchicine 0.5 mg twice daily on regular basis. CRP was 6.09 ± 4.39 (IU/ml) before and 3.34 ± 3.07 (IU/ml) after colchicine. IL-6 was 113.57 ± 37.41 (ng/L) before and 45.57 ± 20.39 (ng/L) after colchicine (*P* = 0.0001).

Whether this significant drop of IL-6and CRP might slow down the rheumatic process and thus ameliorate the valvular lesions is still to be proven. However, it is possible that a better inflammatory status might be translated to clinical benefit especially on long-term follow-up since chronic inflammation appears to have a major role in the progression of valvular damage in patients with RHD [4, 5].

We have previously shown [8] that patients with mitral restenosis after balloon mitral valvuloplasty had higher serum levels of IL-6 and CRP than those who do not have restenosis. This adds to the evidence that progression of valve lesions may be related to higher inflammatory burden. One could wonder about the exact cause of this inflammatory state seen in RHD patients and whether or not genetic factors might play a role at least in the susceptibility and immune response to RF.

Beltrame et al. and more recently Gomaa et al. found that high mannose-binding lectin (MBL) levels increased the risk of RHD and that genotypes associated with high MBL production were associated with both acute and chronic rheumatic carditis. One suggested mechanism was that MBL elicited inflammation and complement tissue damage even in the chronic stage of the disease [9, 10].

Rehman et al. in 2013 studied the role of cytokine gene polymorphisms and their potential usefulness as biomarkers in RHD patients. The study concluded that TNF-α (-308) and IL-6(-174) polymorphisms may be useful markers for the identification of individuals susceptible to RHD. It is plausible that genetics may play a role in perpetuating the chronic inflammatory state seen in patients with RHD. However, further studies are needed to elucidate its precise role [11].

We found that in the three regimens used for RF penicillin prophylaxis, the basic serum level of inflammatory markers was significantly higher in the 30-day regimen than in 15 and 21 days. Moreover, the effect of colchicine on IL-6 and CRP was more marked in the 21- and 15-day than in the 30-day regimen. This might be due to type 1 statistical error due to the small number receiving 30-day regimen. We have recently shown [12] that there is a strong negative correlation between serum levels of inflammatory markers (IL-6 and CRP) and serum penicillin in RHD. The exact mechanism of this finding is unclear. We think it might be related to some anti-inflammatory or anti-streptococcal action of penicillin. Thus, it seems that it is better to use either the 21- or15-day rather than the 30-day regimen of penicillin prophylaxis.

## Conclusion

In our study, we conclude that serum inflammatory markers CRP and IL-6 levels are high in patient with chronic RHD. For the first time, the use of colchicine 0.5 mg twice daily was found to have a significant effect in decreasing the inflammatory markers CRP and IL-6 in patient with chronic RHD. Adding colchicine to the shortest regimen of LAP had better outcome. In fact, the 21- and 15-day regimens are better than the 30-day regimen in ameliorating the chronic inflammatory state in patient with chronic RHD.

## Limitations

1. The study is a single-center study with small number of patients.
2. Only short-term effects of colchicine were studied. Long-term follow-up is needed to assess the effectiveness of anti-inflammatory treatment and possible delay of the progression of valvular fibrosis.

## Data Availability

The datasets used and analyzed during the current study are available from the corresponding author on reasonable request.

## Abbreviations

RHD: Rheumatic heart disease
RF: Rheumatic fever
LAP: Long acting penicillin
IL-6: Interleukin-6
CRP: C-reactive protein
TNF: Tumor necrosis factor
BID: “Bis in die” in Latin means twice daily
MR: Mitral regurgitation
MBL: Mannose-binding lectin

## References

[1] Manyemba J, Mayosi BM (2003). Intramuscular Penicillinis more effective than oral penicillin in secondary prevention of rheumatic fever,a systematic review. S AfrMed J.

[2] Guilherme L, Köhler K, Kalil J (2012) Rheumatic heart disease: genes, inflammation and autoimmunity. Rheumatol Curr Res doi, 10,:1149-1161

[3] Guilherme L, Ramasawmy R, Kalil J (2007). Rheumatic fever and rheumatic heart disease: genetics and pathogenesis. Scand J Immunol.

[4] Davutoglu V, Celik A, Aksoy M (2005). Contribution of selected serum inflammatory mediators to the progression of chronic rheumatic valve disease, subsequent valve calcification and NYHA functional class. J Heart Valve Dis.

[5] Gölbasi Z, Uçar Ö, Keles T, Sahin A, Çagli K, Çamsari A, Diker E, Aydogdu S (2002). Increased levels of high sensitive C-reactive protein in patients with chronic rheumatic valve disease: evidence of ongoing inflammation. Eur J Heart Fail.

[6] Chiu-Braga Y, Hayashi S, Schafranski M, Messias-Reason I (2006). Further evidence of inflammation in chronic rheumatic valve disease (CRVD): high levels of advanced oxidation protein products (AOPP) and high sensitive C-reactive protein (hs-CRP). Int J Cardiol.

[7] Soares ACD, Passos LSA, Sable C, Beaton A, Ribeiro VT, Gollob KJ (2019). Circulating cytokines predict severity of rheumatic heart disease. Int J Cardiol.

[8] Rifaie O, Salem A, Abdel-Rahman M, Raslan H (2014). Does a chronic inflammatory state have a role in the development of mitral restenosis after balloon mitral valvuloplasty?. Int J Cardiol.

[9] Beltrame MH, Catarino SJ, Goeldner I, Boldt ABW, de Messias-Reason IJ (2015). The lectin pathway of complement and rheumatic heart disease. Front Pediatr.

[10] Gomaa MH, Ali SS, Fattouh AM, Hamza HS, Badr MM (2018). MBL2 gene polymorphism rs1800450 and rheumatic fever with and without rheumatic heart disease: an Egyptian pilot study. Pediatr Rheumatol.

[11] Rehman S, Akhtar N, Saba N, Munir S, Ahmed W, Mohyuddin A, Khanum A (2013). A study on the association of TNF-α-308, IL-6-174, IL-10-1082 and IL-1RaVNTR gene polymorphisms with rheumatic heart disease in Pakistani patients. Cytokine.

[12] Rifaie O, Yousef A, Hamza M, Mamin S (2019). Study of the relation of serum long acting penicillin and the inflammatory markers interleukin 6 and C reactive protein in patients with chronic rheumatic heart disease. Eur Heart J.

